# The Importance of COVID-19/Influenza Vaccines Co-Administration: An Essential Public Health Tool

**DOI:** 10.3390/idr14060098

**Published:** 2022-12-05

**Authors:** Roberto Venuto, Ioselita Giunta, Rosaria Cortese, Federica Denaro, Giuseppe Pantò, Antonino Privitera, Smeralda D’Amato, Cristina Genovese, Vincenza La Fauci, Francesco Fedele, Concetta Ceccio, Raffaele Squeri, Alessio Facciolà

**Affiliations:** Department of Biomedical and Dental Sciences and Morphofunctional Imaging, University of Messina, 98125 Messina, Italy

**Keywords:** vaccine co-administration, COVID-19, influenza

## Abstract

Vaccine co-administration is an important tool with several advantages for public health, among which is the increase of vaccination coverage, as well as economic and logistical benefits. The purpose of this study was to assess and compare the immune response to the COVID-19 first booster dose in healthcare workers (HCWs) who chose co-administration and in HCWs who received only COVID-19 vaccination and to investigate personal opinions about the experience of co-administration. We carried out a retrospective analysis involving two groups of HCWs, both vaccinated with the complete primary cycle and the first booster dose of the COVID-19 vaccine, but one of them was also vaccinated, at the same time as the first booster dose, with the influenza vaccine. Active phone calls were also performed, and specific questions about the onset of side effects and general opinions were asked. A good immune response was found in both two groups without any statistically significant difference in the immune response. No severe reactions occurred in either group. A greater part of the sample was completely satisfied, and they would do it again. Our findings are totally in favor of the co-administration, considering the many positive aspects provided by administering, at the same time, more vaccines.

## 1. Introduction

COVID-19 and seasonal influenza are two widespread airborne diseases with a remarkable health impact on people in terms of hospitalizations and deaths [1,2]. Some vulnerable populations, such as the elderly, people with comorbidities and pregnant women, are particularly at risk of obtaining these infections and resulting in negative clinical outcomes [3,4,5,6,7]. For this reason, vaccinating the whole population, especially these fragile people, is one of the main purposes of public health policies. The available vaccines against these two infections are safe and effective; therefore, vaccination programs are the most powerful means in the hands of public health to control both infections [8,9].

As regards the Italian COVID-19 vaccination campaign, fall 2021 was a season characterized by the continuation of the primary cycle doses administration and by the administration of the first and the second booster doses to some categories of individuals, among which are individuals with a high risk of virus exposure, such as healthcare workers (HCWs), people over 60 and people affected by specific pathologies or conditions and defined as “extremely vulnerable” [10]. HCWs have been particularly affected by SARS-CoV-2 during the pandemics, and they were one of the first categories to whom vaccination was carried out with a very high efficacy [11,12].

In the Northern hemisphere, fall is also the season characterized by the influenza vaccination campaign. Influenza vaccination is strongly recommended for people over 60, individuals affected by diseases increasing the risk of influenza complications and HCWs [13]. Therefore, in October 2021, considering the overlapping of the timing of both vaccination campaigns and of the targeted groups to whom both vaccinations are strongly recommended, the Italian Ministry of Health, following the recommendation of the World Health Organization (WHO) and the European Center for Disease Control and Prevention (ECDC), stated that it would have been possible to plan the administration of these two vaccines in the same vaccination session [14,15].

In general, vaccine co-administration can have several advantages: the most important effect is the increase of vaccination coverage, as well as economic and logistical benefits [16,17]. Moreover, about the simultaneous administration of COVID-19 and influenza vaccines, another advantage would be the opportunity to equal or overcome influenza vaccine coverage of the 2020/21 season, in which a very high coverage was reached among the HCWs [18].

Vaccine co-administration is one of the main public health future policies. However, vaccine hesitancy could be a hard obstacle in reaching this purpose. Vaccine hesitancy is a widespread sentiment consisting of a delay in acceptance or refusal of vaccines despite the availability of vaccine services, and fear of side effects is one of the most common determinants [19]. Many people think that administering, at the same time, more vaccines could have a higher impact on the onset of side effects. However, recent scientific evidence showed that co-administration is safe and with a reactogenicity profile similar to that of vaccines administered alone [20].

Our study aimed to assess and compare the serological response one month after the first COVID-19 booster dose, both in HCWs who chose co-administration and in HCWs who received only COVID-19 vaccination. Moreover, we also asked both groups about outcomes and, relative to the group who received both vaccines, personal opinions about this experience.

## 2. Materials and Methods

### 2.1. Sample Collection

The present study was carried out by the Operative Unit of Hospital Hygiene, University Hospital “G. Martino” of Messina, Italy. The vaccination center of the Operative Unit offers all the HCWs the possibility of receiving COVID-19 and influenza vaccines both simultaneously or in a distinct administration. Specifically, in order to study the effects of co-administration in terms of efficacy and safety, we carried out a retrospective analysis involving two groups of HCWs, both composed of 64 people vaccinated with the complete primary cycle and the first booster dose of COVID-19 vaccine but one of them also vaccinated, at the same time of the first booster dose, with influenza vaccine. The only exclusion criterion used for selecting the sample was a previous positivity to COVID-19 to exclude some potential bias linked to the natural immune response.

### 2.2. Sample Processing

The first COVID-19 booster dose was administered to both two groups in a period between 4 and 6 months after the primary cycle. For COVID-19, both cohorts were vaccinated with BNT162b2 COVID-19 mRNA (Pfizer/Biontech). The cohort subjected to co-administration was vaccinated with two different influenza vaccines: the traditional egg-based tetravalent (VAXIGRIP TETRA, Sanofi S.r.l.) and the innovative cell culture-based tetravalent (FLUCELVAX TETRA, Seqirus S.r.l.) flu vaccines. Both the used flu vaccines were updated with the seasonal 2021–2022 composition.

### 2.3. Immune Response Evaluation

In both groups, antibodies against Spike protein were detected after one month from the first booster dose of vaccination to evaluate the efficacy of COVID-19 vaccination and the potential role played by the co-administration with influenza vaccine in the immune response. To this aim, after obtaining informed consent to participate in the study, blood samples were collected and centrifuged at 4,000 rpm for 10 min, and a CLIA (ChemiLuminescence ImmunoAssay) test (LIAISON SARS-CoV-2 S1/S2 IgG—DIASORIN S.p.A., Saluggia, Italia) consisting in a quantitative assay for the detection of IgG antibodies against S1/S2 antigens of SARS-CoV-2 was used. Specifically, values < 0.8 B.A.U./mL were considered negative.

### 2.4. Personal Opinions Evaluation

In order to investigate the effect of the co-administration on the reactogenicity and personal opinions of those receiving both vaccines, active phone calls were performed, and specific questions about the onset of side effects and general opinions were asked.

### 2.5. Statistical Analyses

All the obtained data were collected and analyzed with Prism 4.0 software. Descriptive statistics were used to find percentages, mean values, and standard deviations. The comparison between the study groups was carried out through correlation tests, chi-square tests and Student’s *t*-test. Significance was assessed at the *p* < 0.05 level.

## 3. Results

**Figure 1:**
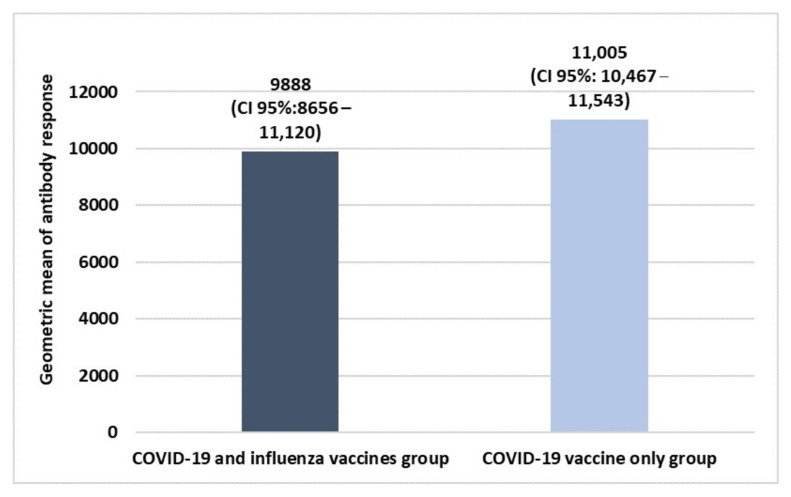
Difference in the immune response to the 1st booster dose of the COVID-19 vaccine between COVID-19 and influenza vaccines and COVID-19 vaccine-only groups. Size sample of both the two groups: 64 HCWs. Student’s *t*-test was used to evaluate statistically significant differences.

The HCWs’ sample co-vaccinated with influenza and COVID-19 vaccines was composed of 64 people, of which 53.1% were men and 46.9% were women, with an average age of 44.1 ± 13.4 (min. 25, max. 67). According to the influenza vaccine, 65.6% of the sample was vaccinated with the egg-based vaccine while 34.4% with the cell culture-based vaccine. The group vaccinated with only the first booster dose of the COVID-19 vaccine was composed of 64 people with the same sex composition of the co-administration group and an average age of 44.2 ± 12.9 (min. 27, max. 67). Figure 1 shows the geometric means of COVID-19 antibody titers in both the two group.

**Figure 2:**
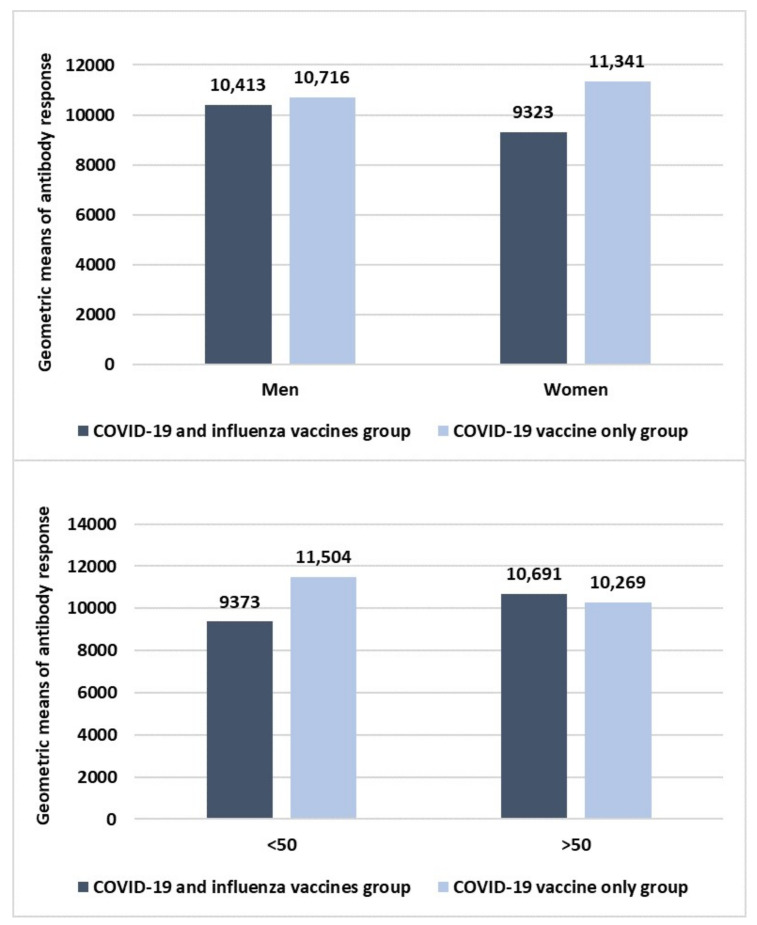
Sex and age differences in the immune response to the first booster dose of the COVID-19 vaccine between COVID-19 and influenza vaccines and COVID-19 vaccine-only groups. Size sample of both the two groups: 64 HCWs. Student’s *t*-test was used to evaluate statistically significant differences.

The figure shows that a good response was found in both the two groups with high values of the geometric mean of antibody response without any statistically significant difference in the immune response between the two groups. Moreover, dividing the data according to sex and age, the results are shown in Figure 2.

According to sex, there was no statistically significant difference between men and women within the groups. Specifically, while men of both the two groups had very similar antibody responses, a certain difference, even if not statistically significant, was found between women of both the two groups with lower antibody response among those who underwent co-administration compared to those vaccinated with the only the first COVID-19 booster dose. Moreover, according to age, no difference was found in those aged >50 years old, while a not statistically significant difference was found for people <50 years old.

**Figure 3:**
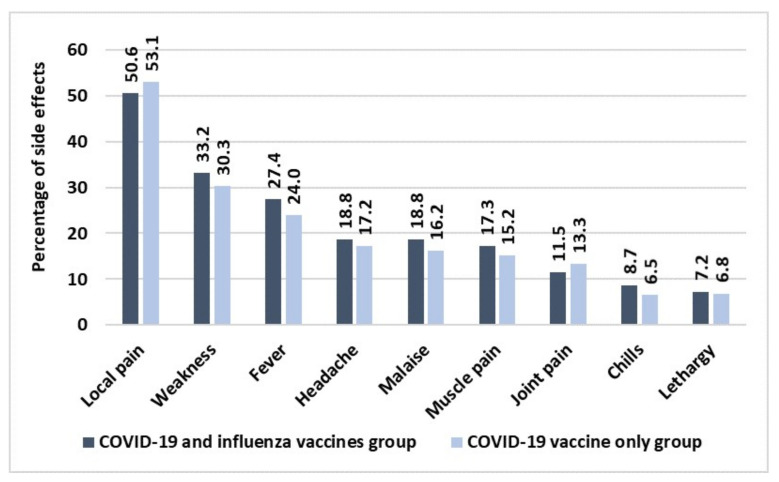
Comparison in the onset of side effects between both the two study groups. Size sample of both the two groups: 64 HCWs.

Concerning the onset of side effects in both groups, no severe reactions occurred in either group but only mild symptoms. The results are shown in Figure 3.

The figure shows that no difference was found in people that received both two vaccinations compared to the COVID-19 vaccine-only group. Further, we considered if some differences in the onset of side effects were present within the co-administration group according to sex and type of flu vaccine used. The results are shown in Table 1.

Finally, questions concerning personal opinions about co-administration and its effects were asked of those that received both vaccines. The results are shown in Table 2.

The table shows that a greater part of the sample was completely satisfied by the co-administration and that they would do it again. Moreover, approximately 8/10 people stated that they would be favorable to a combined flu and COVID-19 vaccine. Some differences, but not statistically significant, were found between sexes, with men that resulted more “repented” than women (8.9% vs. 4.5%) having undergone co-administration. Moreover, women were more inclined than men to do it again (95.5% vs. 84%) and to recommend it (88.6% vs. 82.7%).

## 4. Discussion and Conclusion

Vaccine co-administration may have some potentially positive aspects, including the possibility of reducing public health costs but, above all, the increase of vaccination uptake. This is true, especially for COVID-19 and influenza. Co-administration of COVID-19 and influenza vaccines is a feasible and smart choice. A phase IV randomized placebo-controlled trial established that both ChAdOx1 and BNT162b2 COVID-19 vaccines could be safely co-administered with either MF59-adjuvanted or cell-culture-derived influenza vaccines without any significant increase in adverse events or immunologic interference [21]. Both the WHO and the United States Centers for Disease Control and Prevention (CDC) establish that COVID-19 vaccines can be simultaneously administered with other vaccines, including influenza [14,22]. In Italy, at the beginning of the 2021/2022 influenza vaccination campaign, the Ministry of Health approved the flu vaccine co-administration with other vaccines [23].

The results of our research have shown that a good immunogenicity toward COVID-19 was found in both the study groups without any statistically significant difference between them, even if people vaccinated with only COVID-19 first booster dose had a higher immune response compared to those that underwent co-administration. This finding is in line with some of the previous literature data showing very similar geometric means of antibody response against SARS-CoV-2 in those who underwent a single COVID-19 vaccine compared to those co-vaccinated with flu vaccine [21,24]. Moreover, no statistically significant difference was found according to sex and age.

In addition, people who underwent co-administration did not develop significant side effects compared to those who underwent single first COVID-19 booster dose administration. Side effects were all local and of mild intensity with a very similar percentage to that found in the group subjected to a single administration. In general, COVID-19 vaccines cause above all local side effects represented by pain, erythema and swelling in 10% of vaccinated people. More rarely, some adverse reactions consisting of early local reactions with atypical morphological appearance coinciding with vaccination against COVID-19 have been documented [25]. Although there were no statistically significant differences between the two groups concerning side effects, inside the group who underwent co-administration, some statistically significant differences were found between sexes regarding local pain and weakness with women that resulted in the being more affected than men.

Moreover, the personal opinions stated by the co-vaccinated people were very interesting and stimulating. Specifically, it is to emphasize that almost the totality of the sample did not regret having co-administered, and they would do it again. These findings are very important because, actually, little is known about people’s knowledge, attitudes and practices regarding vaccine co-administration. Stefanizzi et al. [26], in a recent paper, reported a very high frequency (60%) of co-administration of the COVID-19 first booster dose and influenza vaccination among HCWs. This remarkably high co-administration rate was likely due to the particular study population of HCWs who received appropriate and effective information.

Our findings are totally in favor of the co-administration, considering the many positive aspects provided by administering, at the same time, more vaccines; more specifically, the possibility of reducing vaccination sessions and, therefore, the pressure on health facilities. Moreover, because it has been shown that the COVID-19 vaccination schedule was able to interfere negatively with influenza vaccination campaigns during the last season, co-administering the two vaccines could work around this problem. During the 2020/2021 Southern Hemisphere influenza season, the COVID-19 immunization program played a negative role in influenza vaccination uptake. For instance, in Australia, a marked drop in influenza vaccination coverage was reported in all age groups compared to the two previous seasons [27]. However, differently from the 2020/2021 influenza season, during 2021/2022, the above-mentioned clinical guidelines on COVID-19/influenza vaccine co-administration have been issued both by the WHO and the CDC. This fact is crucial because it has been suggested that one of the key actions to improve the acceptance and, therefore, the uptake of COVID-19/influenza vaccines co-administration is the issue of clear, coherent, appropriate and frequent communication on the importance of having both vaccinations by public health authorities [27]. In Italy, a recent survey showed that a very small amount (23%) of the general adult population would be inclined to accept co-administration, while 17% are completely opposed to it. The remaining sample (approximately 60%) results are hesitant to some degree [20]. The main public concern about co-administration can be the fear of potentiating side effects, that too many vaccines overload the immune system and may exert lower efficacy than single vaccines [17]. Many different tools could improve this lack of trust and of knowledge. For instance, the availability and spread of further experimental, observational and pharmacovigilance data and the development of official guidelines issued by scientific associations will probably give a strong impulse to increase public acceptance and the uptake of vaccine co-administration.

In addition, it is to underline that the future of the COVID-19 pandemic and its immunization policies is still unknown, and we cannot exclude the possibility that this infection may turn into a seasonal problem along with influenza. Actually, some evidence has shown how this probability seems more and more likely [28]. In this light, co-administration could be a fundamental tool to reduce the burden on health facilities. Therefore, results like ours may be useful for planning vaccination campaigns in the next seasons.

## Figures and Tables

**Table 1:** Differences in side effects according to sex and type of flu vaccine used.//= not statistically significant.

Differences in Side Effects According to Sex			
	Men (%)	Women (%)	*p* value
Local pain	26.7	43.2	0.017692
Weakness	16.7	29.5	0.030157
Headache	15.0	9.1	//
Fever	16.7	20.5	//
Malaise	13.3	13.4	//
Muscle pain	10.0	13.6	//
Joint pain	5.0	11.4	//
Chills	5.0	6.8	//
Lethargy	3.3	6.8	//
**Differences in side effects according to type of flu vaccine used**			
	Egg-based flu vaccine (%)	Culture cell-based flu vaccine (%)	*p* value
Local pain	39.0	26.7	//
Weakness	22.3	24.4	//
Headache	15.3	8.9	//
Fever	18.6	17.8	//
Malaise	11.9	13.3	//
Muscle pain	11.9	11.1	//
Joint pain	10.1	11.1	//
Chills	5.1	6.7	//
Lethargy	5.1	4.4	//

**Table 2:** Personal opinions about co-administration in the COVID-19 and influenza vaccines group. Size sample: 64 HCWs. Response percentage: 100%.

Questions	Yes (%)	No (%)
Did you regret having co-administered COVID-19 and influenza vaccines?	6.7	93.3
Would you do it again?	89.4	10.6
Would you recommend it?	85.6	14.4
Would you be favourable to a single combined influenza-COVID-19 vaccine?	80.8	19.2

## Data Availability

Not applicable.
